# Different evolutionary dynamics of hepatitis B virus genotypes A and D, and hepatitis D virus genotypes 1 and 2 in an endemic area of Yakutia, Russia

**DOI:** 10.1186/s12879-022-07444-w

**Published:** 2022-05-12

**Authors:** Anastasia A. Karlsen, Karen K. Kyuregyan, Olga V. Isaeva, Vera S. Kichatova, Fedor A. Asadi Mobarkhan, Lyudmila V. Bezuglova, Irina G. Netesova, Victor A. Manuylov, Andrey A. Pochtovyi, Vladimir A. Gushchin, Snezhana S. Sleptsova, Margarita E. Ignateva, Mikhail I. Mikhailov

**Affiliations:** 1 Department of Viral Hepatitis, Russian Medical Academy of Continuous Professional Education, Moscow, Russia 125993; 2 Laboratory of Viral Hepatitis, Mechnikov Research Institute of Vaccines and Sera, Moscow, Russia 105064; 3 Scientific and Educational Resource Center for High-Performance Methods of Genomic Analysis, Peoples’ Friendship University of Russia (RUDN University), Moscow, Russia 117198; 4 JSC «Vector-Best», Research and Production Area, building 36, Koltsovo, Novosibirsk region Russia 630559; 5 N.F. Gamaleya National Research Center for Epidemiology and Microbiology, Ivanovsky Institute of Virology, Ministry of Health of the Russian Federation, Moscow, Russia 123098; 6 Medical Institute, M.K. Ammosov North-Eastern Federal University, Yakutsk, Russia 677010; 7 The Sakha Republic (Yakutia) Regional Department of Rospotrebnadzor, Yakutsk, Russia 677027; 8 Medical Faculty, Belgorod State National Research University, Belgorod, Russia 308015

**Keywords:** Hepatitis B virus, Hepatitis delta virus, Viral genotypes, Vaccination, Phylodynamics, Evolutionary dynamics

## Abstract

**Background:**

The geographic distribution of the hepatitis B virus (HBV) and the hepatitis D virus (HDV) genotypes is uneven. We reconstructed the temporal evolution of HBV and HDV in Yakutia, one of the regions of Russia most affected by HBV and HDV, in an attempt to understand the possible mechanisms that led to unusual for Russia pattern of viral genotypes and to identify current distribution trends.

**Methods:**

HBV and HDV genotypes were determined in sera collected in 2018–2019 in Yakutia from randomly selected 140 patients with HBV monoinfection and 59 patients with HBV/HDV. Total 86 HBV and 88 HDV genomic sequences isolated in Yakutia between 1997 and 2019 were subjected to phylodynamic and philogeographic Bayesian analysis using BEAST v1.10.4 software package. Bayesian SkyGrid reconstruction and Birth–Death Skyline analysis were applied to estimate HBV and HDV population dynamics.

**Results:**

Currently, HBV-A and HDV-D genotypes are prevalent in Yakutia, in both monoinfected and HDV-coinfected patients. Bayesian analysis has shown that the high prevalence of HBV-A in Yakutia, which is not typical for Russia, initially emerged after the genotype was introduced from Eastern Europe in the fifteenth century (around 600 (95% HPD: 50–715) years ago). The acute hepatitis B epidemics in the 1990s in Yakutia were largely associated with this particular genotype, as indicated by temporal changes in HBV-A population dynamics. HBV-D had a longer history in Yakutia and demonstrated stable population dynamics, indicating ongoing viral circulation despite vaccination. No correlation between HBV and HDV genotypes was observed for coinfected patients in Yakutia (r = − 0.016069332). HDV-2b circulates in Russia in Yakutia only and resulted from a single wave of introduction from Central Asia 135 years ago (95% HPD: 60–350 years), while HDV-1 strains resulted from multiple introductions from Europe, the Middle East, Central Asia, and different parts of Russia starting 180 years ago (95% HPD: 150–210 years) and continuing to the present day. The population dynamics of HDV-1 and HDV-2 show no signs of decline despite 20 years of HBV vaccination. The Birth–Death Skyline analysis showed an increase in the viral population in recent years for both HDV genotypes, indicating ongoing HDV epidemics.

**Conclusions:**

Taken together, these data call for strict control of HBV vaccination quality and coverage, and implementation of HBV and HDV screening programs in Yakutia.

**Supplementary Information:**

The online version contains supplementary material available at 10.1186/s12879-022-07444-w.

## Background

Hepatitis B is a serious global healthcare problem. Despite a highly effective vaccine which has been available for decades, the hepatitis B virus (HBV) is still prevalent in many regions of the world, resulting in 296 million cases of chronic infection globally [1]. According to WHO estimates, HBV-associated mortality in 2015 reached almost 900,000 deaths, which were mainly associated with the long-term outcomes of chronic infection, such as liver cirrhosis and hepatocellular carcinoma (HCC) [2].

HBV is classified into ten genotypes, HBV-A to HBV-J, differing from each other by 7.5–15% at the nucleotide level of complete genomes [3]. Nearly 40 sub-genotypes with 4–7.5% divergence of the entire genomic sequence, named with the genotype letter followed by a digit, are recognized [4]. Genotypes have a distinct geographical distribution, with HBV-A prevalent in Europe, North America, Southeast Africa, and India; HBV-B and HBV-C in Asia and Oceania; HBV-D, the most widespread, in North America, North Africa, Europe, the Middle East, and Oceania; HBV-E in West Africa; HBV-F in South America; and HBV-G and HBV-H in Central and South America [5]. Different HBV genotypes may be associated with different clinical outcomes and prognosis of treatment response [6–10]. In addition, the HBV genotype can also influence the outcome of hepatitis delta [11], since hepatitis delta virus (HDV) infection is possible only in the presence of HBV, as HBV surface proteins are required for the HDV virion envelope to form [12]. In areas where several HBV genotypes are prevalent, such as South America, HBV genotypes D and F have been shown to be associated with higher HDV viral load compared to HBV genotype A in coinfected patients [13].

In turn, HDV is also classified in genotypes (HDV-1 to HDV-8) based on complete genome sequence similarity > 80% [14]. HDV-1 predominates globally, while other genotypes have more limited geographic distribution, with HDV-2 prevalent in Asia, HDV-3 in Latin America (predominating in the Amazon basin), HDV-4 in Japan and China (Taiwan), HDV-5 in Western Africa, and HDV-6 to HDV-8 in Central Africa [15]. Chronic HDV/HBV coinfection leads to accelerated progression of liver disease compared to HBV monoinfection [16]. Data on clinical significance of HDV genotypes are limited. Although HDV-1 is considered to be the most pathogenic [17], HDV-3 is associated with an increased risk of fulminant hepatitis [18], while HDV-2 is less frequently associated with fulminant hepatitis in acute infection and has slower progression to cirrhosis or HCC in chronic infection as compared to HDV-1 [19].

Very little is known about the possible correlation between different HBV and HDV genotypes in coinfected patients, although some epidemiological studies and data from experiments in vitro suggest possible preferences in different HDV/HBV genotype combinations [13, 20].

In Russia, genotypes HBV-D and HDV-1 are predominant [21–23]. However, in one particular region of Russia, Yakutia, HBV-A and HBV-D are both prevalent [24]. Similarly, HDV-1 and HDV-2 are both prevalent in this region [25]. HDV-2 sequences from Yakutia are distinct from other HDV-2 strains which originated in Southeast Asia and are allocated to the separate sub-genotype HDV-2b [23]. Thus, the unique HBV and HDV patterns in Yakutia are completely different from those observed in other parts of Russia.

Yakutia is a large region spanning areas of Central and Northern Siberia. Hepatitis B and D, and the associated outcomes, are a serious healthcare problem in this region [26], as HBV prevalence in Yakutia was three or four times higher than the average in Russia in the pre-vaccination period [27]. Following the 1998 implementation of universal newborn vaccination throughout Russia, including Yakutia, the reported incidence of acute hepatitis B in Yakutia dropped to 0.2–0.4 per 100,000 in recent years. However, the annual rates of chronic hepatitis B are still high (24.9 per 100,000 in 2019), [28] indicating the elevated HBV burden in the region.

The reasons for the unusual distribution of the HBV and HDV genotypes in Yakutia, as well as the possible relationship between genotypes of the two viruses in coinfected patients, have not been studied. The aim of this study was to reconstruct the temporal evolution of HBV and HDV in Yakutia in an attempt to understand the possible mechanisms that led to this pattern of viral genotypes and to identify current distribution trends.

## Methods

### Serum samples

Serum samples from randomly selected 140 patients with chronic HBV monoinfection and 59 patients with chronic HBV/HDV coinfection were collected in 2018–2019 in Yakutia. Written informed consent was obtained from all participants. The whole study was conducted in accordance with the principles expressed in the World Medical Association Declaration of Helsinki regarding ethical medical research involving human subjects. The study design was approved by the Ethics Committee of the Mechnikov Research Institute for Vaccines and Sera in Moscow, Russia (Approval #1 dated February 28, 2018).

All serum samples were transported to lab at the Mechnikov Research Institute for Vaccines and Sera using a cold chain and stored in aliquots at − 70 °C before testing.

### HBV and HDV amplification, sequencing, and genotyping

Viral nucleic acids were extracted from the patients’ sera using the QIAamp Viral RNA Mini Kit (QIAGEN, Hilden, Germany). HDV complete genome sequences were amplified with primers, resulting in two overlapping fragments described by Celik et al. [29]. using the Transcriptor First Strand cDNA Synthesis Kit and Fast Start High Fidelity PCR System (Roche Applied Science, Mannheim, Germany), according to the manufacturers’ protocols. All amplicons were excised from an agarose gel and subjected to nucleic acid isolation using a QIAquick Gel Extraction Kit (QIAGEN, Hilden, Germany) according to the manufacturer’s protocol, and sequenced on the Ion Torrent S5 platform. Libraries were prepared using the Ion 510 & Ion 520 & Ion 530 Kit-Chef (Thermo Fisher Scientific, Waltham, USA) for up to 200 base-read libraries. All samples were read with a coverage of at least 500×, and quality not lower than Q30. The read assembly was performed by BWA-MEM. HDV sequences from this study were deposited in GenBank under the following accession numbers: OK142820–OK142858, OK142920–OK142929, OK142938, and OK142939. Additionally, HDV R0 fragment was amplified using protocol describe elsewhere [30] from 22 patient samples in which the complete genome amplification failed. These 22 R0 sequences were sequenced using the 3500 Genetic Analyzer (ABI, Foster City, USA) and BigDye Terminator v 3.1 Cycle Sequencing Kit according to the manufacturer’s protocol and deposited in GenBank under the following accession numbers: OL875352-OL875373. The HDV genotype was determined based on phylogenetic analysis with the reference HDV sequences recommended in the ICTV 2018 classification [31] using the maximum likelihood (ML) method in the MEGA 7.0.18 software [32].

HBV amplification was performed in two stages. First, HBV DNA testing was performed in an in-house PCR assay with nested primers for overlapping regions of S and P genes adapted from Basuni and Carman [33] with a detection limit of about 50 IU/ml based on the results of serial dilution testing for PCR standards with known viral load. The resulting 713 bp amplicons were extracted from the gel as described above and subjected to direct Sanger sequencing on the 3130 Genetic Analyzer (ABI, Foster City, USA) automatic sequencer using the BigDye Terminator v3.1 Cycle Sequencing Kit according to the manufacturer’s protocol.

Next, we amplified complete HBV genomic sequences with primers using a protocol described elsewhere [22] for 13 HBV-monoinfected patients whose serum samples had an available remaining volume > 500 ml for DNA extraction using MagNA Pure Compact Nucleic Acid Isolation Kit I Large Volume (Roche Applied Science, Mannheim, Germany). Amplification was performed using the Expand™ Long Template PCR System (Roche Applied Science, Mannheim, Germany). Libraries were prepared from amplicons using Qiagen FX DNA Library kit (Qiagen, Hilden, Germany) according to the manufacturer’s protocol and were sequenced on the Illumina Miseq 250PE platform. Complete HBV genomic sequences have been deposited in GenBank under the following accession numbers: OK143470, OK143474, OK143477, OK143478, OK143480, OK143482, OK143485, OK143489, OK143490, OK143494-OK143496, and OK143498.

The HBV genotype was determined based on phylogenetic analysis of a 650 nucleotide fragment of the S-gene (nucleotide positions 157–806 by reference sequence NC_003977.2) with the references recommended by the ICTV [3] and suggested for HBV sub-genotype designation [4] using the ML method in the MEGA 7.0.18 software [32].

As the majority of patients with HBV/HDV coinfection did not have detectable HBV viremia, the HBV genotype was predicted for these 59 patients based on the results of HBsAg serotyping in the ELISA kit (Vector-Best, Novosibirsk, Russia) according to the manufacturer’s protocol. Briefly, HBsAg testing was performed in parallel with three different monoclonal antibody-based conjugates allowing differentiation between the following HBV serotypes/genotypes: ayw2 or ayw3/D, adw2/A, adrq+/C.

### Time-scaled phylogenetic analysis

Complete HDV genomic sequences from this study were supplemented with 327 sequences from GenBank for which the year and country of isolation were known, and which had a divergence of at least 1%. HBV analysis incorporated complete genomic sequences from this study and an additional 321 sequences from GenBank, including ancient sequences obtained from mummies. Several filters were applied for both datasets (skipredundant with a cutoff of 1%) to remove overabundant sequences from each location. In resulting datasets, the ratio between Russian isolates and sequences from databases was at least 1:1.5, and for sequences from Yakutia this ratio was at least 1:5.

The alignment for both HBV and HDV datasets was performed using the ClustalW algorithm in the MEGA 7 software [32]. The most appropriate substitution model was estimated using Jmodeltest-2.1.10. The most suitable models were determined to be SYM+G for the HDV dataset and GTR+G for HBV, which were chosen because they had the lowest Bayesian information criterion (BIC) value. The presence of a positive correlation between genetic divergence and sampling time for HBV and HDV was checked using TempEst v.1.5 software. This analysis also made it possible to calculate the initial clock rates for HBV and HDV (Additional file 1: Fig. S1). The phylogenetic trees were built using the PhyML-3.1 software.

Bayesian analysis was performed using the BEAST v1.10.4 software package. After trial runs to determine the optimal parameters, the SRD06 nucleotide substitution model with a strict clock was selected as the optimal model for HDV. The size was constant as the coalescent tree prior was used. All sequences were checked to ensure reading frame accuracy; no stop codons were identified. The base frequencies were estimated. The initial clock rate 2.74*10^−3^ subs./site/year was used to estimate a clock rate. The Markov Chain Monte Carlo (MCMC) method was run for 50 million generations and sampled every 5000 steps in two repetitions.

Similar work was carried out for HBV, but the parameters of the final runs were as follows: Yang96 nucleotide substitution model, lognormal relaxed clock, Bayesian Skyline Plot (BSP) 5 group. The initial clock rate 1.18*10^−5^ subs./site/year was used to estimate a clock rate [34]. Likewise, the MCMC method was run for 50 million generations and sampled every 5000 steps in two repetitions.

For both viruses the two parallel runs were combined using LogCombiner v1.10. 4. Tracer v1.6 was used to check convergence. The effective sample size was > 500 in both cases. Trees were annotated with TreeAnnotator v.1.10.4 using a burn-in of 1000 trees, and visualized with FigTree v.1.4.3.

### Skyline analysis for reconstruction of HBV and HDV population dynamics

The Skyline methods were used to extract data on the population dynamics of genotypes HBV-A and HBV-D, and HDV-1 and HDV-2 in Yakutia from phylogenetic trees. For this purpose, we used trees that were built only on the sequences that were collected in Yakutia. The vast majority of HBV and HDV sequences from Yakutia that were available from previous studies were restricted to genome fragments, representing the R0 region for HDV and the S-gene for HBV. Consequently, we limited the Skyline analysis to sequences, corresponding to a 379 nucleotide fragment of HDV R0 region (nucleotide positions 899–1279 by reference sequence M21012) and a 650 nucleotide fragment of HBV S-gene (nucleotide positions 157–806 by reference sequence NC_003977.2), respectively. For this purpose, 15 HDV sequences and 73 HBV sequences isolated in Yakutia between 1997 and 2016 by various researchers were added to sequences obtained in this study (Additional file 1: Table S1). The resulting datasets comprised 29 sequences for HBV-A, 57 for HBV-D, 44 for HDV-1, and 44 for HDV-2.

Two different methods, Bayesian SkyGrid reconstruction and Birth–Death Skyline analysis, were applied to four datasets, representing HBV-A, HBV-D, HDV-1, and HDV-2, respectively. For all datasets, the main parameters for both models were taken from calculations based on primary trees built with all reference sequences. The Bayesian SkyGrid reconstruction was performed using BEAST v1.10.4 software. In all cases, the Bayesian SkyGrid coalescent model was used with the Tree Prior parameter defined as equal to 50 and the final time point of 100 years before the most recent sampling. The MCMC method was run for 100 million generations and sampled every 1000 steps. Tracer v1.7.2 was used for visualization.

A Birth–Death Skyline analysis was performed to calculate the reproduction number (Re) separately for HBV-A and HBV-D genotypes, and HDV-1 and HDV-2 genotypes, respectively, using the BEAST2 software. The length of the MCMC was set to 100 million generations. R-package bdskytools was used to visualize the results and construct plots.

### Statistical analysis

Data analysis was performed using graphpad.com. Statistical analysis included assessment of the significance of differences in values between groups using the Fisher exact test (significance threshold p < 0.05). Differences for quantitative values were assessed using Student’s *t*-test (significance threshold p < 0.05).

## Results

### HBV and HDV genotypes in monoinfected and coinfected patients

The distribution of HBV and HDV genotypes among patients with monoinfection and coinfection is shown in Table 1. The median age in the study cohort was 44 years (16–82 years), and the male-to-female ratio was 1:1.15. In patients with HBV monoinfection, viral genotypes were determined based on the results of Sanger sequencing, as well as HDV genotypes in patients with coinfection. As HBV DNA was undetectable in the majority of samples from coinfected patients [42/59 (71.2%)], we used the ELISA kit to determine HBV genotype based on HBsAg serotyping. To validate this technique, we first applied this method to samples from 82 patients with HBV monoinfection from this study, whose viral genotypes were determined by Sanger sequencing. In 81 out of 82 samples (98.8%), the HBV genotype was identified correctly by the ELISA kit. In one sample with the HBV-C genotype, determined by sequencing, a discordant result was obtained using ELISA (HBV-A, serotype adw2). The detailed results of the comparison of the genotyping methods are shown in Additional file 1: Table S2. The strong correlation between the results obtained using sequencing and those from ELISA allowed direct comparison of the data on HBV genotyping obtained in monoinfected and coinfected patients by these two methods. No significant differences were observed in the prevalence of either of the predominant genotypes (HBV-A and HBV-D) between groups of monoinfected and coinfected patients (Table 1).

**Table 1:** Distribution of viral genotypes among HBV-monoinfected and HBV/HDV-coinfected patients in Yakutia

Patient cohort	Number of patients	HBV-A n (% [95% CI])	HBV-D	HBV-C	HDV-1	HDV-2
HBV-monoinfected^a^	140	51 (36.4% [28.9–44.7])	82 (58.6% [50.3–66.4])	7 (5.0% [2.3–10.1])	–	–
HBV/HDV-coinfected^b^	59	29 (49.2% [36.8–61.6])	30 (50.1% [38.4–63.2])	0 (0% [0.0–7.3])	32 (54.2% [41.7–66.3])	27 (45.8% [33.7–58.3])
p value, Fisher exact test		0.1138	0.3497	n.d.	0.4616	

*n.d.* not determined, *95% CI* 95% confidence interval

^a^HBV genotypes were determined by Sanger sequencing

^b^HBV genotypes were deduced from HBsAg serotype determined in ELISA

Patients with HBV-A were significantly younger than patients with HBV-D and HBV-C (median age 37 years vs. 51 years and 57 years, respectively, p < 0.05, Student test), but the sex ratio was similar (1:1 vs. 1:1.3 and 1:1.3, respectively).

The distribution of HDV-1 and HDV-2 in coinfected patients was almost equal, at 54.2% and 45.8%, respectively. No significant differences in patients’ median age or sex ratio were observed between the two HDV genotypes. The distribution of genotypes HBV-A and HBV-D among patients infected with HDV-1 and HDV-2 was uniform (Table 2). No correlation was observed between HBV and HDV genotypes in coinfected patients, with correlation coefficient r = − 0.016069332.

**Table 2:** Distribution of HBV genotypes among patients with HBV/HDV coinfection

Viral genotypes	HBV-A, n (%)	HBV-D, n (%)
HDV-1, n = 32	16 (50%)	16 (50%)
HDV-2, n = 27	13 (48.2%)	14 (51.8%)

### Reconstruction of the history of HBV and HDV circulation in Yakutia

Prior to Bayesian phylogenetic analysis, we looked for a temporal signal in the HBV and HDV datasets, i.e. whether there were genetic changes between sampling time points sufficient to produce a statistically significant relationship between genetic divergence and time. The linear regression curves were observed for both datasets (Additional file 1: Fig. S1), indicating the presence of a positive correlation between genetic divergence and sampling time.

**Fig. 1:**
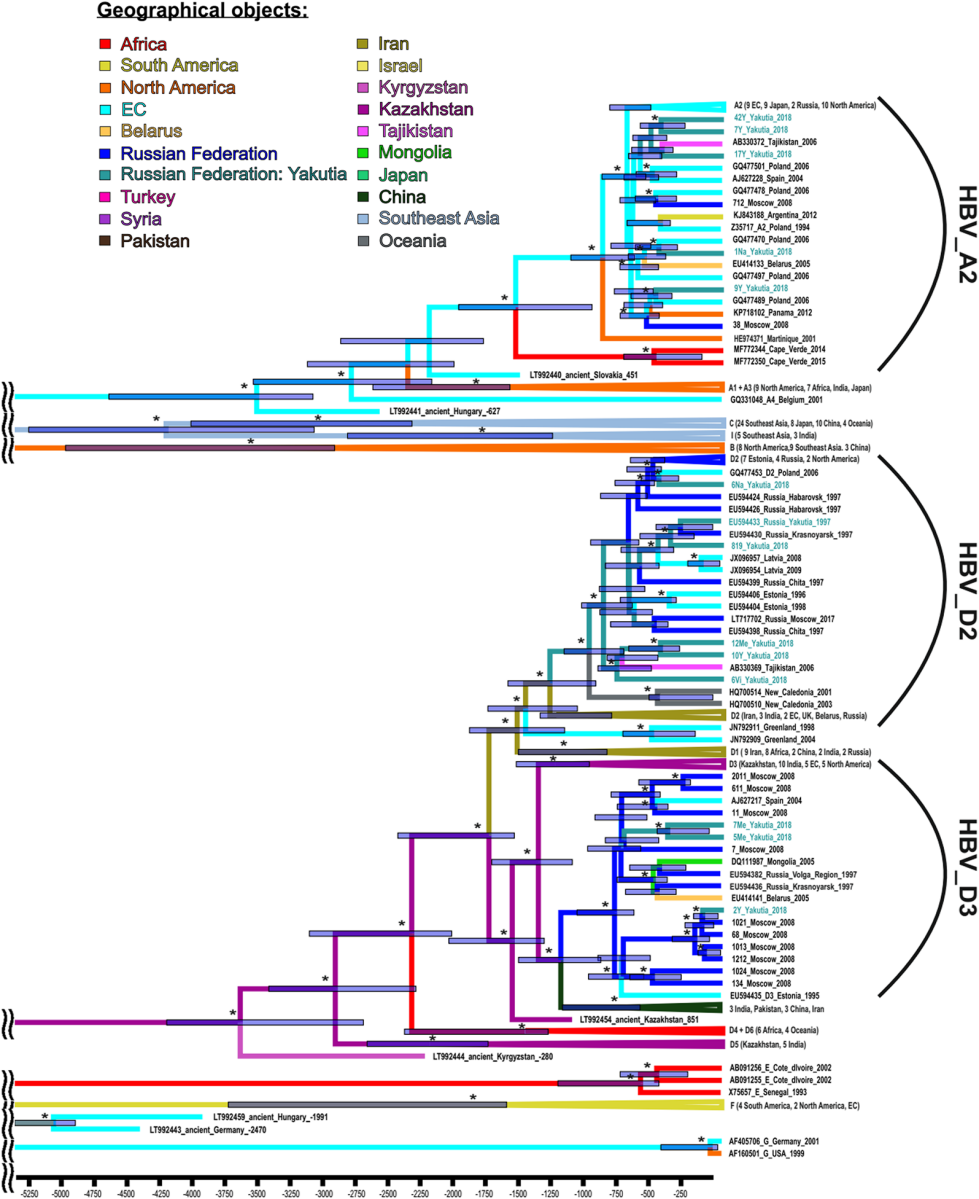
Bayesian phylogenetic tree based on complete genome HBV sequences. The tree root was cut off to ensure the visibility of the modern parts of the tree. Tree nodes with posterior probability > 90% are marked with asterisk. For each sequence, the number in the GenBank database, the HBV genotype, the country, and the year of isolation are indicated. Sequence names from the samples collected for this study are shown in turquoise. For compressed clusters, the number of sequences and regions of isolation are given in parenthesis. The colors of tree branches represent the places of introduction and origin. In each tree node, the 95% HPD is shown as a gray bar. The *X* axis shows time in years presented descending from the date of collection of the most recent sample

A Bayesian phylogenetic tree for HBV is shown in Fig. 1. We were unable to amplify complete HBV genomic sequences from any sample from HBV/HDV coinfected patients, perhaps due to low HBV viral load in these samples. Thus, phylogenetic tree in Fig. 1 contains HBV sequences from this study obtained from monoinfected patients only. The phylodynamic analysis demonstrated that HBV sub-genotype D3, which we observed in Yakutia, had an extensive distribution throughout Russia. This variant is believed to have entered Russia 840 years ago (95% HPD: 691–1128 years) and have diverged about 1253 years ago (95% HPD: 946–1577 years) from the ancestor of the strains that are currently common in India, China, Pakistan, and Iran. The whole group can be traced back to the HBV variant found in ancient graves in Kazakhstan (remains dated to 851 AD). The second HBV genotype D variant from Yakutia belongs to the D2 sub-genotype that is also prevalent in different regions of Russia. This sub-genotype was first introduced to Yakutia around 1036 years ago (95% HPD: 768–1227 years). All HBV-A sequences from Yakutia belong to sub-genotype A2 and are grouped into several clusters with strains of different origin, mainly from Belarus and Poland at a node age of around 560 years (95% HPD: 499–699 years).

**Fig. 2:**
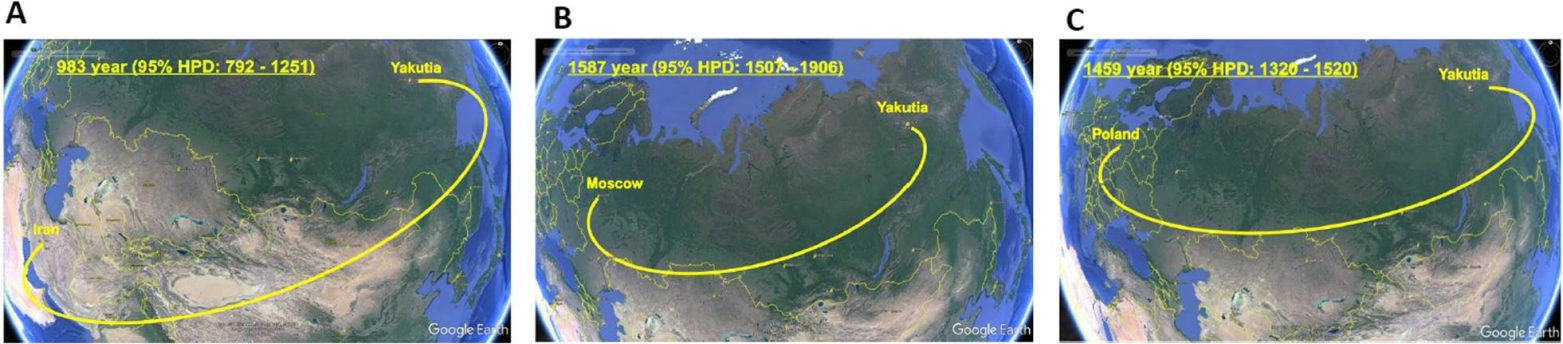
Phylogeographic dispersion of HBV-D2 (**A**), HBV-D3 (**B**) and HBV-A (**C**) waves of introduction in Yakutia. The spatial phylogenetic reconstruction of the evolutionary dynamics traced in the study is shown in the map referring to the region of study. Yellow dispersion lines indicate HBV introduction to Yakutia. The years of introduction are shown with 95% HPD interval

Phylogeographic analysis demonstrated that HBV-D, namely sub-genotype D2, was first introduced into Yakutia from the territory of modern Iran in the tenth century (Fig. 2A), while sub-genotype D3 was imported from European part of Russia much later, in sixteenth century (Fig. 2B). HBV-A was imported in the fifteenth century from the territory of modern Poland (Fig. 2C). Yakut strains served as a source of further HBV-A spread in Russia.

**Fig. 3:**
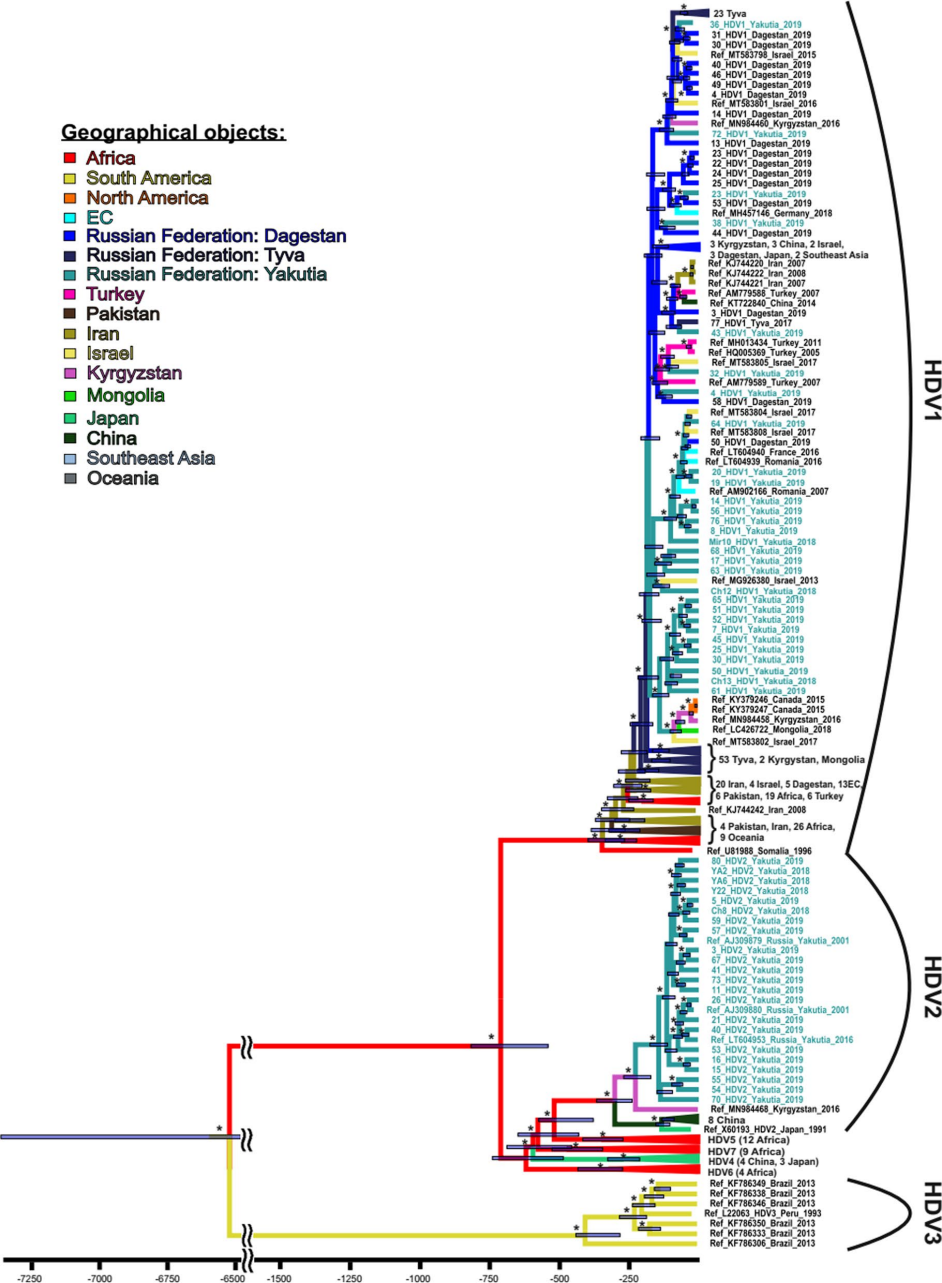
A Bayesian phylogenetic tree based on HDV complete genome sequences. Tree nodes with posterior probability > 90% are marked with asterisk. For each sequence, the number in the GenBank database, the HDV genotype, the country, and the year of isolation are indicated. Sequence names from the samples collected for this study are shown in turquoise. For compressed clusters, the number of sequences and regions of isolation are given. The colors of tree branches represent the places of introduction and origin. In each node the 95% HPD is shown as a gray bar. The *X* axis shows time in years presented descending from the date of collection of the most recent sample

A Bayesian phylogenetic tree for HDV is shown in Fig. 3.

The history of HDV circulation in Yakutia is much shorter than that of HBV evolution in the region. HDV-1 has a long history of being introduced to this region, starting 180 years ago (95% HPD: 150–210 years) and continuing to the present day. These introductions were all of Eastern European or Middle Eastern origin, coming from Turkey, Romania, and Israel. Often, variants of this genotype circulated first in Russia, in Dagestan or Tuva, and were only later introduced to Yakutia. All HDV-2 sequences from Yakutia belong to the 2b sub-genotype and form a monophyletic group, suggesting the existence of the “founder effect”, i.e. resulting from a single wave of introduction. Since it was introduced 135 years ago (95% HPD: 60–350 years), genotype HDV-2 has spread widely throughout Yakutia, but has not gone beyond this region, as it has not been detected anywhere else in Russia so far. Contrary to previous belief that the HDV-2 variant came to Yakutia from East Asia, our data demonstrate the presence of an intermediate ancestor, the closest “descendant” of which is now found in Kyrgyzstan.

### Analysis of HBV and HDV population dynamics

To assess the population dynamics for HBV and HDV in Yakutia, we performed SkyGrid reconstruction for sequences obtained in this particular region. The number of complete genome sequences obtained at different time points in Yakutia is very limited. To verify whether the smaller genome fragments were informative, we first performed analysis in parallel for complete genomic sequences and their most frequently sequenced fragments (S-gene for HBV and R0 for HDV). The graphs illustrating trends in the effective number of infections over time were similar when constructed based on HBV complete genome sequences and a 650 nucleotide fragment of S-gene, with HPD values comparable for both plots throughout the last 40 years (Additional file 1: Fig. S2). A similar effect was observed for HDV when comparing graphs illustrating the trends in the effective number of infections over time for complete genome sequences and the R0 fragment (Additional file 1: Fig. S3). Thus, for the SkyGrid reconstructions of population dynamics that were performed separately for genotypes HBV-A, HBV-D, HDV-1, and HDV-2, we supplemented the respective datasets with available sub-genomic sequences obtained in Yakutia between 2001 and 2018.

**Fig. 4:**
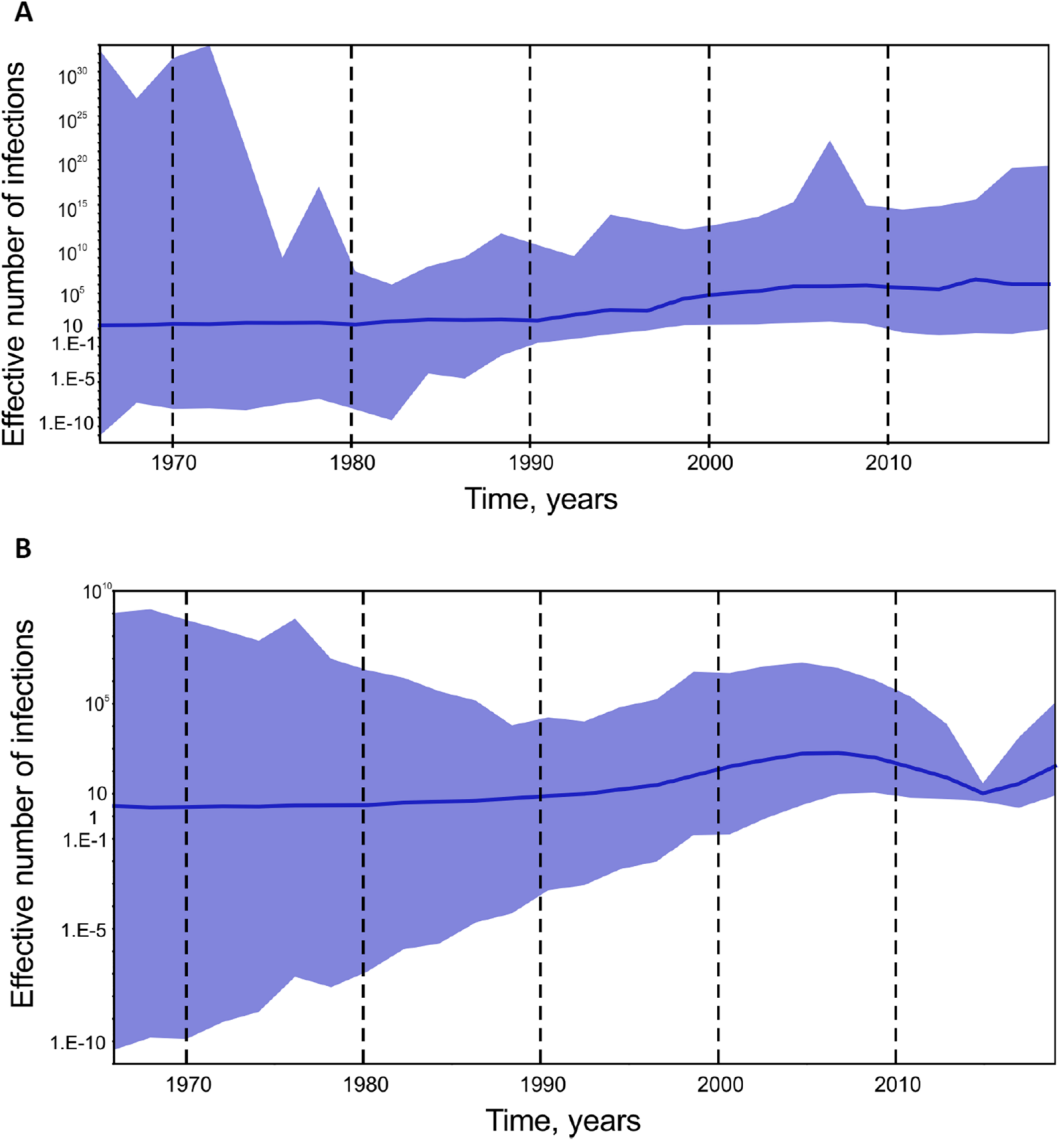
SkyGrid reconstruction for HBV-A (**A**) and HBV-D (**B**). The graphs show the relationship between the effective number of infections (*y* axis) and the chronological time expressed in years (*x* axis). The blue curve indicates the mean, and the 95% HPD interval is shown by the blue filling. Estimates were obtained using 29 HBV-A and 57 HBV-D sequences of a 650 nucleotide fragment of HBV S-gene from Yakutia

Analysis of the effective number of HBV-A and HBV-D infections in Yakutia demonstrated the start of slow exponential growth in the 1990s for both viral genotypes (Fig. 4). However, the magnitude of the predicted effective number of infections was several decimal logarithms higher for HBV-A (Fig. 4A) compared to HBV-D (Fig. 4B). In addition, HBV-D showed a decline in the number of infections after 2010 with a subsequent rebound in recent years.

**Fig. 5:**
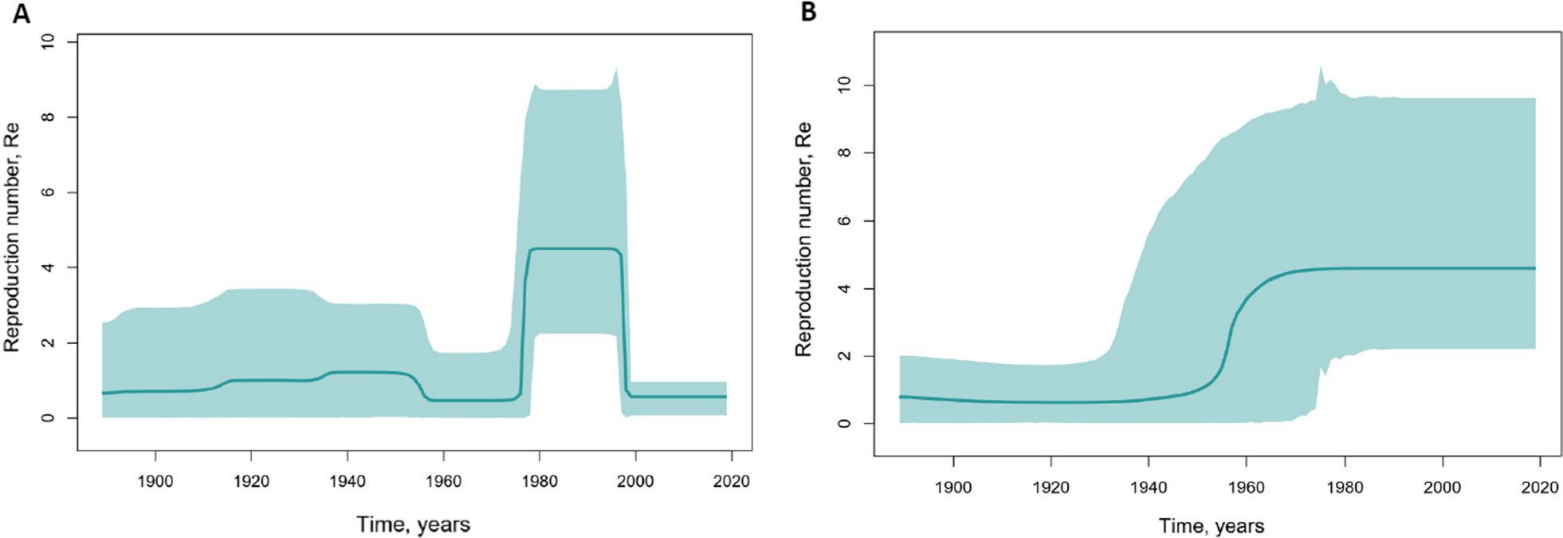
Birth–Death Skyline reconstruction for HBV-A (**A**) and HBV-D (**B**). The graphs show the relationship between the reproduction number of infections (*y* axis) and the chronological time expressed in years (*x* axis). The curves indicate the mean, and the 95% HPD interval is shown by the green filling. Estimates were obtained using 29 HBV-A and 57 HBV-D sequences of a 650 nucleotide fragment of HBV S-gene from Yakutia

The changes in the reproduction number (Re) over time, indicating the number of new infections from one source and predicted using Birth–Death Skyline analysis, showed significant differences in the population dynamics of genotypes HBV-A and HBV-D in Yakutia (Fig. 5).

Re values for HBV-A increased rapidly around 1980, peaked throughout the 1980s and 1990s, and rapidly declined in the 2000s (Fig. 5A). HBV-D demonstrated exponential growth in Re values during the 1950s. The values reached a plateau in the 1960s and have since remained stable (Fig. 5B). A Death Skyline analysis performed separately for sub-genotype D2 and D3 sequences from Yakutia showed the same pattern for these HBV-D sub-genotypes (Additional file 1: Fig. S4).

**Fig. 6:**
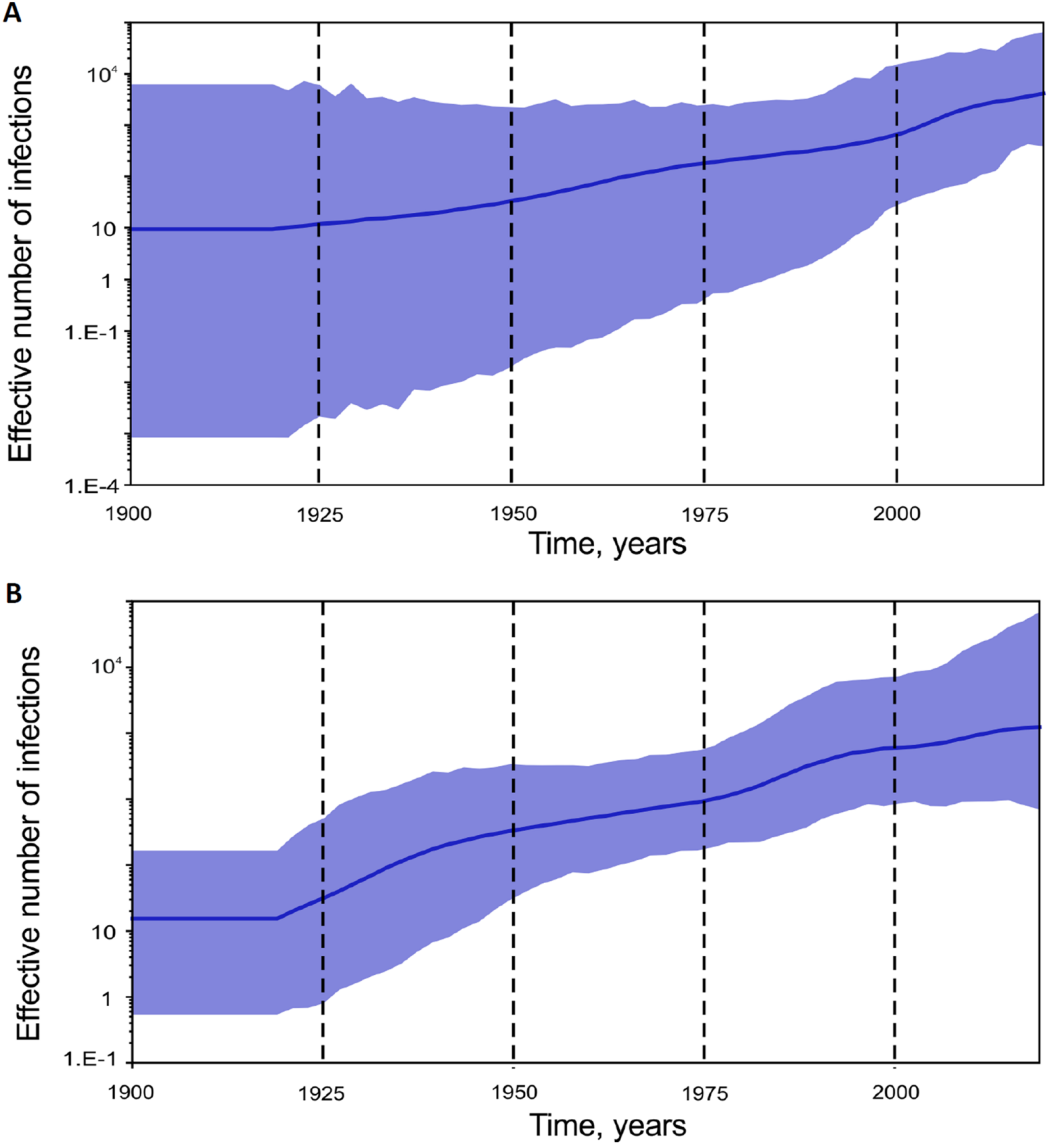
SkyGrid reconstruction for HDV-1 (**A**) and HDV-2 (**B**). The graphs show the relationship between the effective number of infections (*y* axis) and the chronological time expressed in years (*x* axis). The blue curve indicates the mean, and the 95% HPD interval is shown by the blue filling. Estimates were obtained using 44 HDV-1 and 44 HDV-2 sequences of a 379 nucleotide fragment of HDV R0 region from Yakutia

SkyGrid reconstruction analysis for HDV-1 and HDV-2 (Fig. 6) showed that slow exponential growth of both viral genotypes began in the 1920s and continued until recent years. Growth in the effective number of infections over time was similar for HDV-1 and HDV-2, although HDV-2 showed more intense growth in the 1920s to the 1950s (Fig. 6A) compared to HDV-1 (Fig. 6B), and unlike HDV-2, genotype HDV-1 had maintained exponential growth by 2020.

**Fig. 7:**
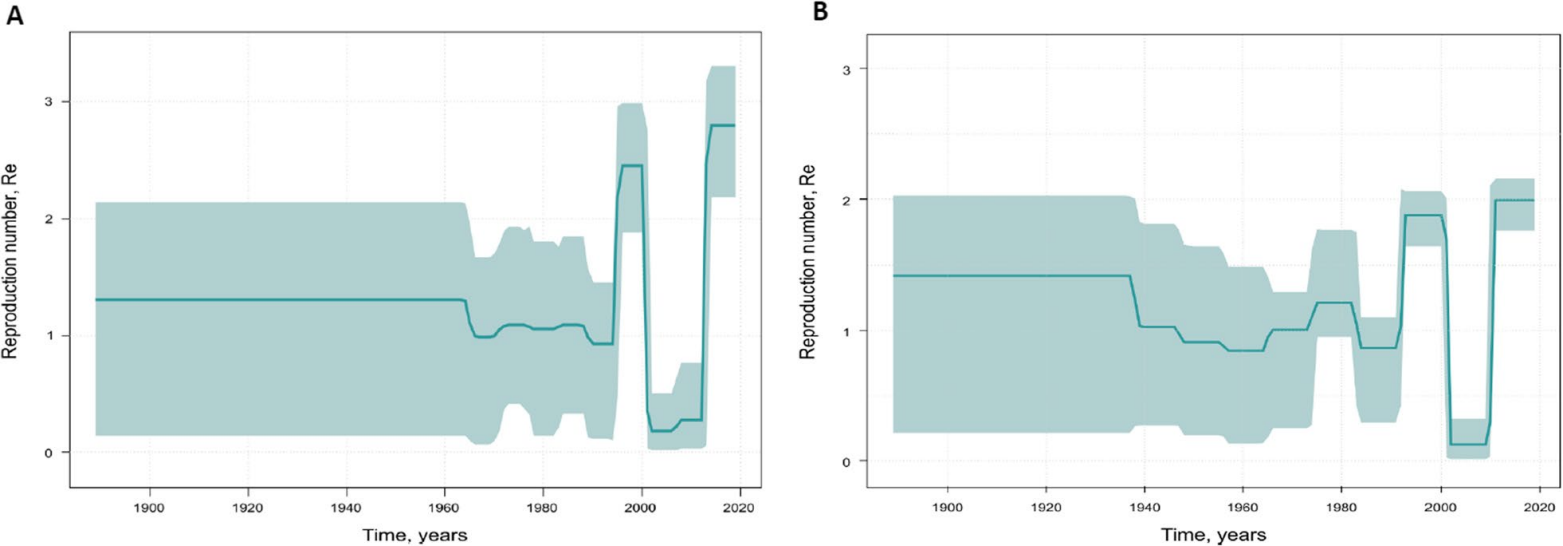
Birth–Death Skyline reconstruction for HDV-1 (**A**) and HDV-2 (**B**). The graphs show the relationship between the reproduction number of infections (*y* axis) and the chronological time expressed in years (*x* axis). The curves indicate the mean, and the 95% HPD interval is shown by the green filling. Estimates were obtained using 44 HDV-1 and 44 HDV-2 sequences of a 379 nucleotide of HDV R0 region from Yakutia

Birth–Death Skyline analysis highlighted more complex population dynamics for HDV-1 and HDV-2 in Yakutia (Fig. 7). The predicted Re values for HDV-1 remained stable until the 1990s (Fig. 7A), while the Re for HDV-2 experienced an increase around the 1980s (Fig. 7B). Since the early 1990s, both genotypes showed a very similar trend in Re values over time, with a sharp rise in the 1990s, followed by a drop to below 0.5 after 2000, and a further sharp increase in recent years.

## Discussion

In this paper, we have described the molecular epidemiology of HBV and HDV in Yakutia, one of the regions of Russia most affected by these infections. We have shown that HBV-A and HBV-D are both predominating genotypes in Yakutia, with HBV-C being a minor HBV genotype. In general, this is consistent with the previous literature on the subject [24]. However, we observed some changes in the HBV-A and HBV-D ratio compared to the 2008 report. In our study, the proportion of HBV-A decreased from 63 to 36% and, conversely, the proportion of HBV-D increased from 37 to 58%.

We also showed that two HDV genotypes, HDV-1 and HDV-2, are still widespread in Yakutia. Moreover, our data indicate that HBV-A and HBV-D are equally common in HDV-coinfected patients, with no specific preference in HBV genotypes for HDV-1 and HDV-2 viruses. These data suggest that both HDV genotypes can use HBsAg of the HBV-A or HBV-D viruses with approximately the same probability and/or efficiency. The possible limitation of this results is the lack of HBV sequences from HDV/HBV coinfected patients, as in this particular cohort HBV genotype was predicted based in HBsAg serotyping results. However, the validation study demonstrated that HBV genotype was assigned correctly in 98.8% by the ELISA kit, suggesting the correct HBV genotyping results in vast majority of samples from coinfected patients.

Although the unusual distribution of HBV and HDV genotypes in Yakutia has previously been studied, no attempts have been made to explain this phenomenon. We used Bayesian analysis to reconstruct the history of HBV and HDV circulation in Yakutia. The most difficult point in any phylogenetic reconstruction of the history of viral evolution is a lack of sequences that are old enough to make the analysis reliable. The publication in recent years of ancient HBV sequences isolated from mummies and remains dating from the Bronze Age to the Middle Ages [34–36] allows more reliable temporal estimates of the evolution of this virus to be made.

Time-scaled phylogeographic analyses have shown that HBV-D, namely sub-genotype D2, was prevalent in Yakutia for centuries, while HBV-A (sub-genotype A2), was introduced to the region for the first time from Eastern Europe in the fifteenth century. This is consistent with the history of the colonization of Siberia. Interestingly, the Skyline analysis demonstrated a significant rise in the reproduction number for HBV-A in the 1990s, suggesting that the increase in the epidemic was associated with this particular genotype. Unlike HBV-A, genotype HBV-D had no peak in its reproduction number during this period, suggesting distinct transmission patterns for these HBV genotypes. The differences in the main transmission routes for different HBV genotypes or sub-genotypes are well documented. For example, sub-genotype A1 transmission is perinatal, while A2 is mainly acquired by adults parenterally or sexually [5]. Patients with HBV-A were significantly younger than HBV-D patients in our study, suggesting the possible differences in transmission patterns. The rise in HBV-A population dynamics in the late 1990s in Yakutia coincided with peak acute hepatitis B incidence in this region at 36.6 per 100,000 by 1997 [37]. At the very same time, the highest estimates of the proportion of intravenous drug users, up to 40%, were reported among patients with acute hepatitis B [38]. Taken together, these data suggest that the rise in HBV-A population dynamics in Yakutia was associated with HBV epidemics in intravenous drug users in the 1990s, similar to the massive spread of the hepatitis C virus genotype 3a observed around this time in the former Soviet Union [39]. In addition, our data indicate that the reproduction number calculated based on Skyline analysis may be a sensitive indicator of dynamic changes in HBV epidemiology.

The stable HBV-D population dynamics observed in our study despite ongoing HBV vaccination over the last 20 years is noteworthy. Indeed, long-term HBV vaccination is expected to result in a decrease in viral genetic diversity and population size. However, we were not able to observe such a decrease in Yakutia, despite a significant decline in acute hepatitis B incidence in Yakutia, which was only 0.2–0.4 per 100,000 in 2017–2019 [28]. Recently, we have shown ongoing HBV circulation in Yakutia in children born after the vaccination program was rolled out, with HBsAg positivity rates as high as 4% in children aged 10–14 years old and a 10% anti-HBc positivity rate in the same group [40]. This continued HBV circulation, possibly associated with HBV-D, may be attributed to vaccination gaps or/and failures such as faults in the vaccine cold chain, and is consistent with the stable HBV-D population dynamics observed in this study.

We have shown that HDV, unlike HBV, began to spread in Yakutia relatively recently, no more than 200 years ago. HDV-1 was introduced to the region many times from European and Central Asian countries, as well as from other regions of Russia, for example from Tuva. These data suggest that Yakutia was part of a global HDV-1 epidemic process. At the same time, HDV-2 came to Yakutia only once, apparently from Central Asia, leading to the so-called “founder effect”, i.e. all Yakut HDV-2 sequences form a monophyletic group. It remains unclear why HDV-2 did not spread to neighboring regions of Russia. It is possible that the low population density and relatively low degree of mobility of the population in the northeastern regions of Russia was a factor.

Unfortunately, there are no ancient HDV sequences because it is an RNA virus. This is an important limitation encountered in all reconstructions of HDV evolutionary history. Nevertheless, the available HDV sequences make it possible to reconstruct the history of the evolution of HDV over the past decades, with a rather small 95% HPD, which is very important for understanding viral circulation against the background of HBV vaccination. Thus, using Bayesian analysis, Nogueira-Lima et al. demonstrated a decrease in the epidemiological levels of HDV-3 in South America after the introduction of HBV vaccination and donor screening [41]. Interestingly, our data on HDV epidemiological dynamics in Yakutia do not indicate any stable decrease after the implementation of newborn HBV vaccination in 1998, either in the effective number of infections or in the reproduction number. Moreover, in this regard, both genotypes, HDV-1 and HDV-2, behave the same: after the initial drop in calculated reproduction numbers at the beginning of the 2000s, these values have returned to pre-vaccination era levels in recent years, suggesting ongoing HDV epidemics among HBV-infected patients, of whom there are many in this region. In this regard, the highly informative value of the reproduction number should be noted, as it offered a better reflection of possible changes in the HBV and HDV epidemiological process than the number of effective infections, although the latter also reflects the main trends.

Interestingly, the rise in the reproduction number observed both for HDV-1 and HDV-2 in the 1990s coincided with an increase in the same indicator for HBV-A. Consequently, we can assume that the first wave of HDV epidemics may have been largely associated with HBV-A and resulted from increasing intravenous drug use. A similar epidemic rise in HBV/HDV coinfection associated with HBV-D and HDV-1 was documented in young patients, mainly intravenous drug users, in the late 1990s in Samara, a city in the European part of Russia [42].

The recent increase in HDV population dynamics against a background of stable HBV-D population dynamics suggests ongoing silent HDV epidemics despite very low reported incidence of acute hepatitis B. These data indicate that although current HBV prevention measures, such as newborn HBV vaccination and donor screening, are quite effective in preventing new cases of HBV infection, they are not sufficient to combat HDV epidemics in Yakutia. Expanded screening programs and mandatory HDV testing for all patients diagnosed with hepatitis B are necessary to assess the true HDV burden in this region.

## Conclusion

The temporal changes in HBV-A evolutionary dynamics suggest that the acute hepatitis B epidemics in the 1990s in Yakutia were largely associated with this particular genotype. Unlike HBV-A, HBV-D demonstrated stable population dynamics, indicating ongoing viral circulation despite vaccination. No correlation in the HBV-infecting genotype was observed for HDV-1 and HDV-2 genotypes in Yakutia. In Russia, HDV-2 circulates only in Yakutia, and resulted from a single wave of introduction from Central Asia in the nineteenth century, while HDV-1 strains resulted from multiple introductions from Europe, the Middle East, Central Asia, and different parts of Russia. The population dynamics of HDV-1 and HDV-2 show no signs of decline despite 20 years of HBV vaccination. Moreover, Skyline analysis showed an increase in viral population in recent years for both HDV genotypes, indicating ongoing HDV epidemics. Taken together, these data demonstrate the need for strict control of HBV vaccination quality and coverage, and implementation of HBV and HDV screening programs in Yakutia.

## Supplementary Information

**Additional file 1: Table S1.** Information on HBV and HDV sequences from Yakutia that were used for Skyline analysis. **Table S2.** Comparison of HBV genotyping results obtained using Sanger sequencing and ELISA kit for HBsAg serotyping. **Figure S1.** Temporal signal linear regression graphs for HBV (a) and HDV (b) complete genomic sequences. The X-axis shows time in years. **Figure S2.** SkyGrid reconstruction for HBV complete genome (a) and HBV S-gene 650 nt fragment (b) datasets. **Figure S3.** SkyGrid reconstruction for HDV complete genome (a) and 379 nt fragment of HDV R0 region (b) datasets. **Figure S4.** SkyGrid reconstruction for HBV subgenotypes D2 (a) and D3 (b).

## Data Availability

All data generated or analyzed during this study are included in this article and its Additional file 1.

## Abbreviations

HBV: Hepatitis B virus
HDV: Hepatitis D virus
HCC: Hepatocellular carcinoma
HBsAg: Hepatitis B surface antigen
CI: Confidence interval
HPD: Highest Posterior Density
MCMC: Markov Chain Monte Carlo
