# Supplementary material for: Different evolutionary dynamics of hepatitis B virus genotypes A and D, and hepatitis D virus genotypes 1 and 2 in an endemic area of Yakutia, Russia
Source: BMC Infect Dis. 2022 May 12;22:452. doi: 10.1186/s12879-022-07444-w (PMC9097355; doi:10.1186/s12879-022-07444-w)
Supplement: Supplementary file 1 — Additional file 1: Table S1. Information on HBV and HDV sequences from Yakutia that were used for Skyline analysis. Table S2. Comparison of HBV genotyping results obtained using Sanger sequencing and ELISA kit for HBsAg serotyping. Figure S1. Temporal signal linear regression graphs for HBV (a) and HDV (b) complete genomic sequences. The X-axis shows time in years. Figure S2. SkyGrid reconstruction for HBV complete genome (a) and HBV S-gene 650 nt fragment (b) datasets. Figure S3. SkyGrid reconstruction for HDV complete genome (a) and 379 nt fragment of HDV R0 region (b) datasets. Figure S4. SkyGrid reconstruction for HBV subgenotypes D2 (a) and D3 (b). [file 12879_2022_7444_MOESM1_ESM.docx]

**Additional table S1.** Information on HBV and HDV sequences from Yakutia that were used for Skyline analysis

| Virus | Year of isolation | Number of sequences | GenBank accession number |
| --- | --- | --- | --- |
| HBV | 2018 | 13¹ | OK143470, OK143474, OK143477, OK143478, OK143480, OK143482, OK143485, OK143489, OK143490, OK143494-OK143496, OK143498 |
|  | 2014 | 35 | KM212957, KP143742-KP143745, KP165597-KP165605, KP184495-KP184495, KP202936-KP202945, KP230541, KT962021-KT962025 |
|  | 2008 | 8 | OL771254-OL771261 |
|  | 2004-2006 | 16 | OM025238-OM025253 |
|  | 1997 | 14 | AY653781, AY653782, AY653787-AY653789, AY653796, AY653799, EU594390-EU594395, EU594433 |
| HDV | 2018-2019 | 73¹ | OK142820–OK142858, OK142920–OK142929, OK142938, OK142939, OL875352-OL875373 |
|  | 2016 | 1 | LT604953 |
|  | 2001 | 2 | AJ309879, AJ309880 |
|  | 2001 | 12 | AJ309868-AJ309878, AJ309881 |

¹ Sequences obtained in this study

**Additional table S2.** Comparison of HBV genotyping results obtained using Sanger sequencing and ELISA kit for HBsAg serotyping

| HBV genotype determined by sequencing | Number of tested samples | HBV serotype and deduced genotype by ELISA | Number of correctly identified samples | Concordance, % |
| --- | --- | --- | --- | --- |
| HBV-A | 46 | adw2, HBV-A | 46 | 100% |
| HBV-D | 32 | ayw2 or ayw3, HBV-D | 32 | 100% |
| HBV-C | 4 | adrq+, HDV-C – in 3 samples;  adw2, HBV-A –  in 1 sample | 3 | 75% |
| All HBV genotypes combined | 82 | - | 81 | 98.8% |

| 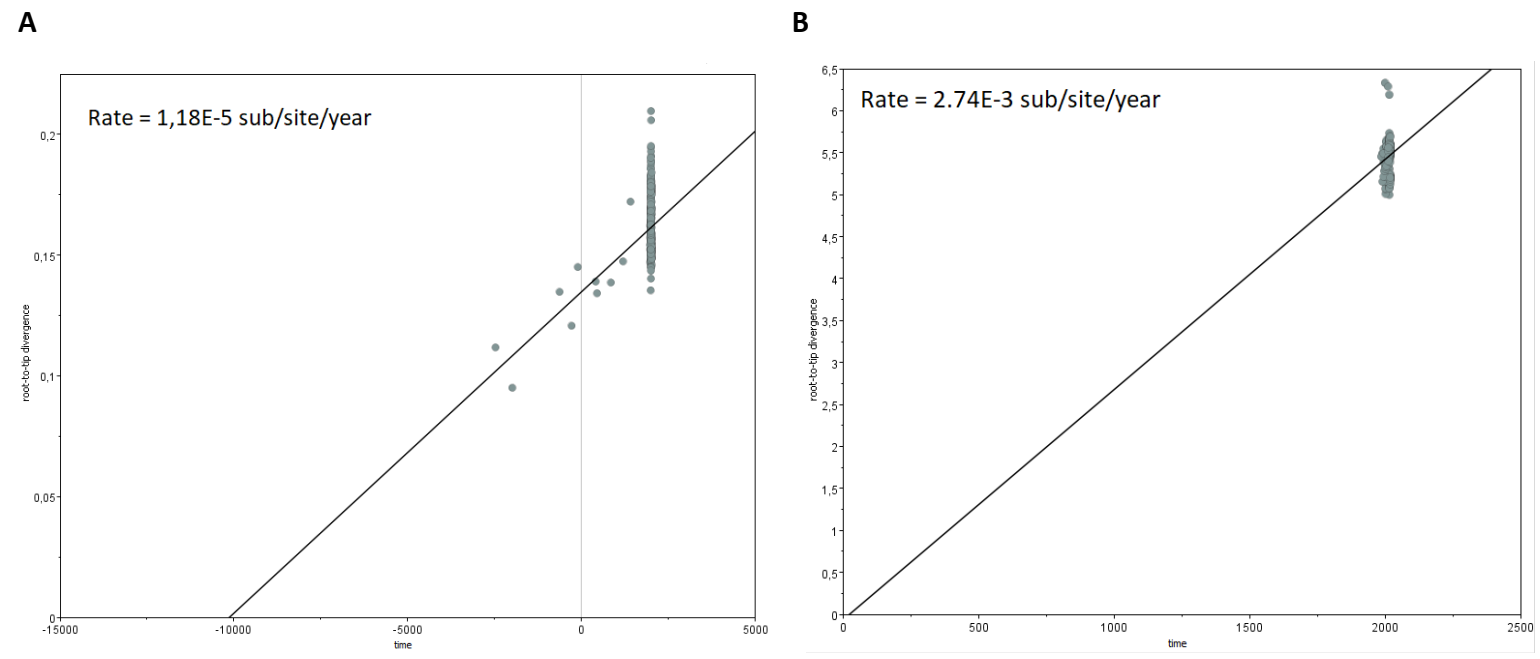 | 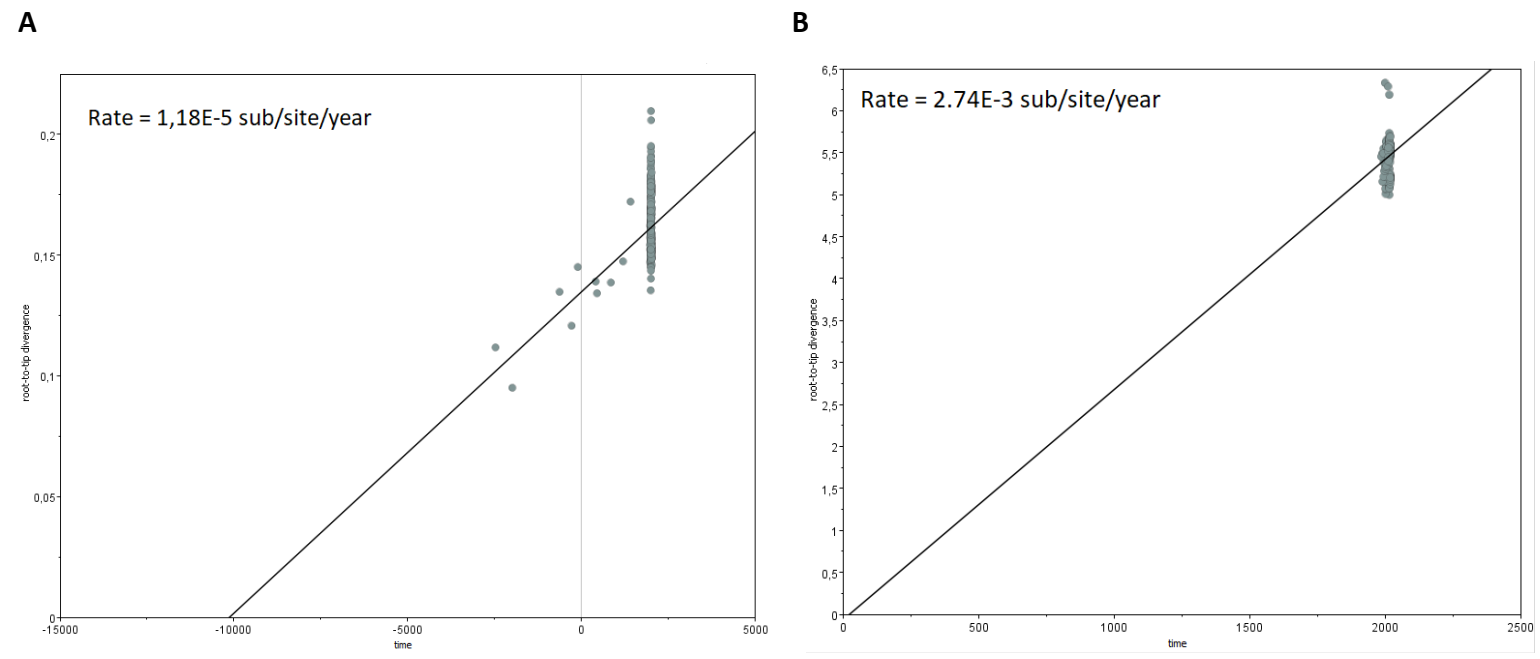 |
| --- | --- |
| (**a**) | (**b**) |

**Additional figure S1.** Temporal signal linear regression graphs for HBV (a) and HDV (b) complete genomic sequences. The X-axis shows time in years.


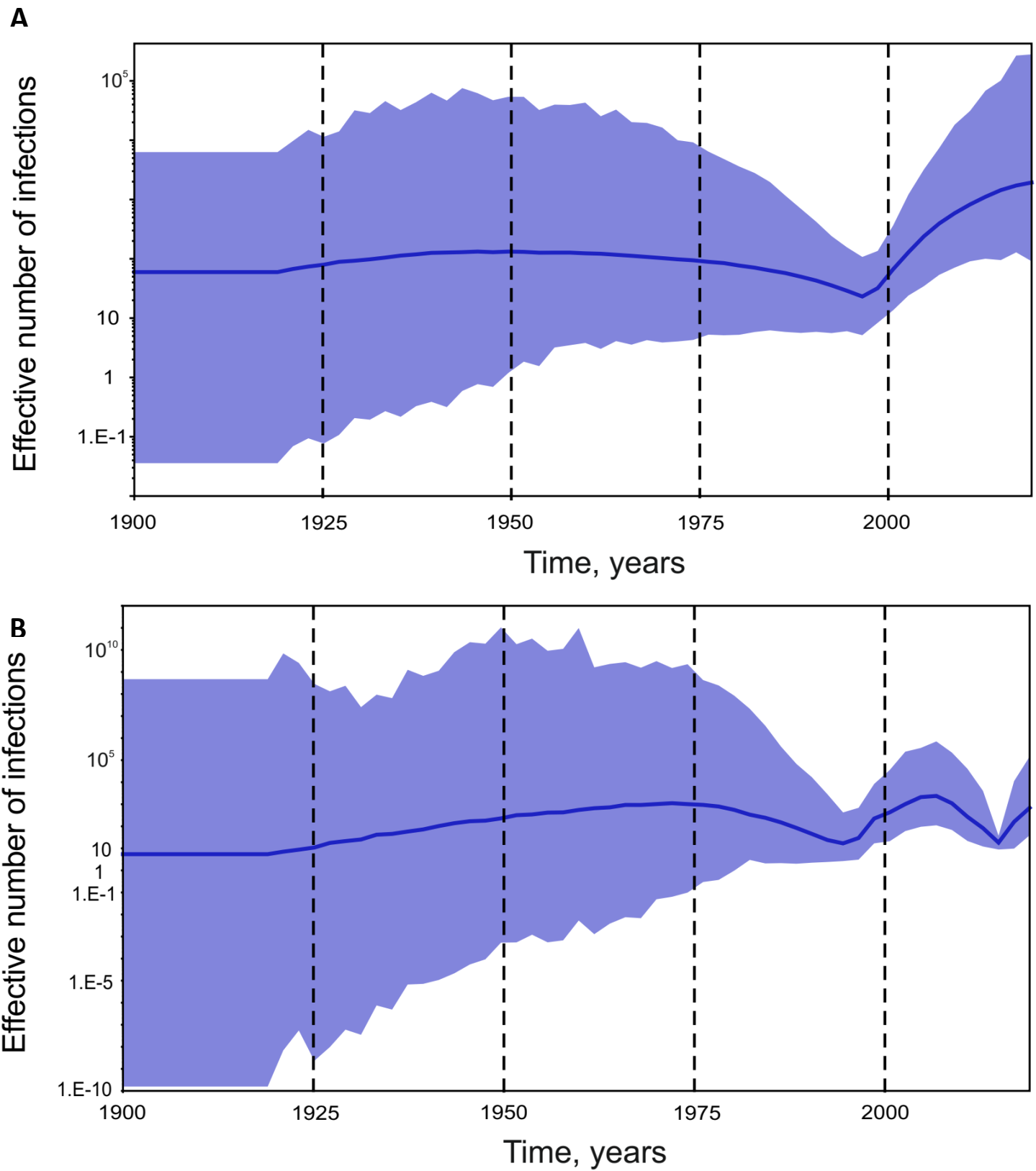


**Additional figure S2.** SkyGrid reconstruction for HBV complete genome (a) and HBV S-gene 650 nt fragment (b) datasets. The graphs show the relationship between the effective number of infections (y-axis) and the chronological time expressed in years (x-axis). The blue curve indicates the mean, and the 95% HPD interval shown in lilac filling.

| 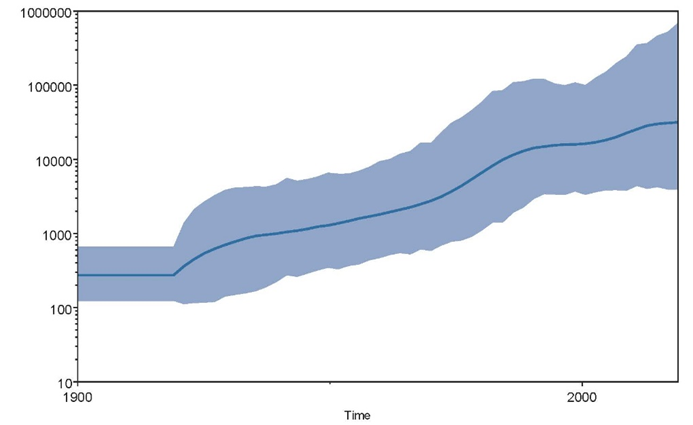 | 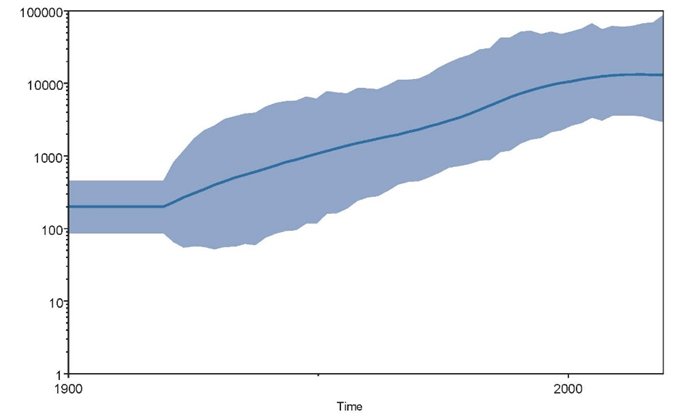 |
| --- | --- |
| (**a**) | (**b**) |

**Additional figure S3.** SkyGrid reconstruction for HDV complete genome (a) and 379 nt fragment of HDV R0 region (b) datasets. The graphs show the relationship between the effective number of infections (y-axis) and the chronological time expressed in years (x-axis). The blue curve indicates the mean, and the 95% HPD interval shown in blue filling.

| 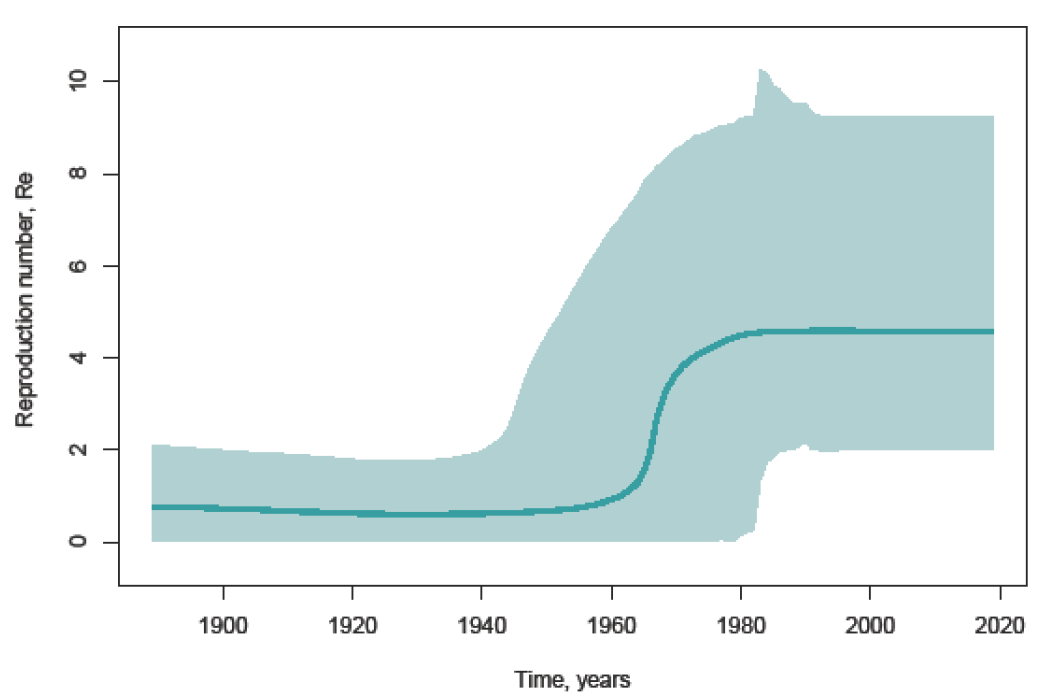 | 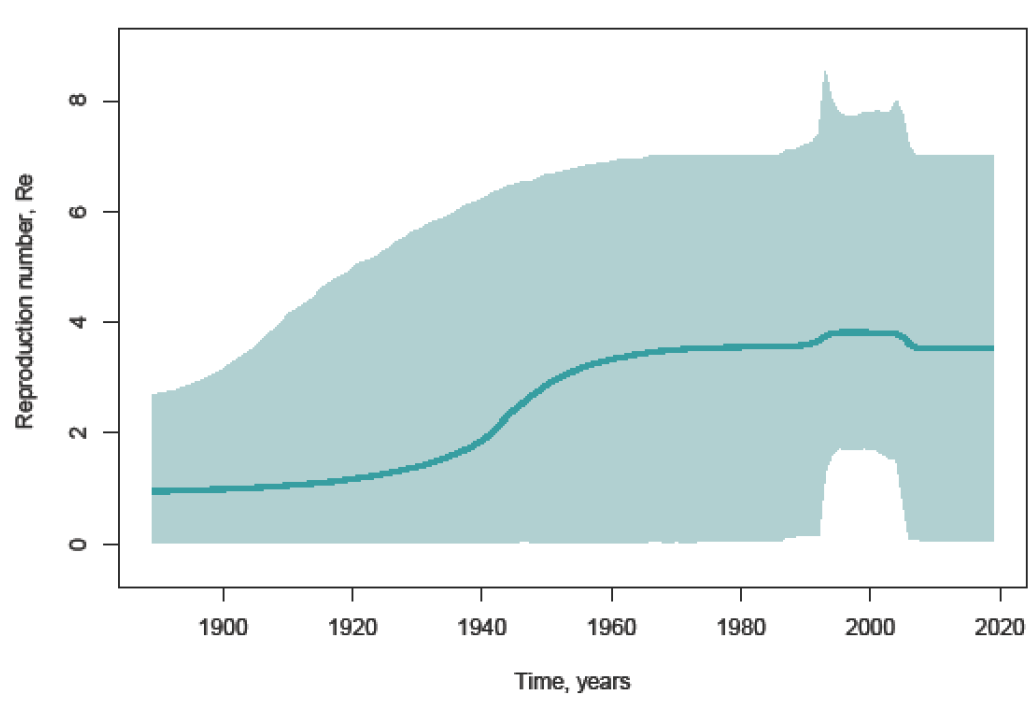 |
| --- | --- |
| (**a**) | (**b**) |

**Additional figure S4.** SkyGrid reconstruction for HBV subgenotypes D2 (a) and D3 (b). The graphs show the relationship between the effective number of infections (y-axis) and the chronological time expressed in years (x-axis). The green curve indicates the mean, and the 95% HPD interval shown in green filling.
